# High-dimensional spatiotemporal visual analysis of the air quality in China

**DOI:** 10.1038/s41598-023-31645-1

**Published:** 2023-04-04

**Authors:** Jia Liu, Gang Wan, Wei Liu, Chu Li, Siqing Peng, Zhuli Xie

**Affiliations:** School of Space Information, Space Engineering University, Beijing, 101416 People’s Republic of China

**Keywords:** Environmental impact, Climate-change impacts, Climate-change policy, Sustainability

## Abstract

Air quality is a significant environmental issue among the Chinese people and even the global population, and it affects both human health and the Earth’s long-term sustainability. In this study, we proposed a multiperspective, high-dimensional spatiotemporal data visualization and interactive analysis method, and we studied and analyzed the relationship between the air quality and several influencing factors, including meteorology, population, and economics. Six visualization methods were integrated in this study, each specifically designed and improved for visualization analysis purposes. To reveal the spatiotemporal distribution and potential impact of the air quality, we designed a comprehensive coupled visual interactive analysis approach visually express both high-dimensional and spatiotemporal attributes, reveal the overall situation and explain the relationship between attributes. We clarified the current spatiotemporal distribution, development trends, and influencing factors of the air quality in China through interactive visual analysis of a 25-dimensional dataset involving 31 Chinese provinces. We also verified the correctness and effectiveness of relevant policies and demonstrated the advantages of our method.

## Introduction

In China, the acceleration of urbanization has driven the development of industrialization and centralization of society and the economy. At the same time, the excessive use of land and natural resources has caused various serious ecological degradation and environmental pollution problems, threatening both human health and sustainable urban development. One of the more prominent environmental pollution problems is air pollution. Frequent haze weather has caused notable concern among people^1^, has significantly and persistently impacted all aspects of society, and has become a hot issue concerning the public, government, and academia^2^.

The world's largest population can be found in China, which encompassed a land area of 9.6 million square kilometer. By the end of 2021, the urban population reached 914.25 million people, the rural population amounted to 498.35 million people, and the gross domestic product (GDP) reached 114,366.97 billion yuan, of which industrial production accounted for 39.4% of the total amount. From 20.5317 million units in 2002 to 294.1859 million units in 2021, the number of civilian automobiles has grown. Several dangerous substances can be found in vehicle exhaust, including harmful gases, suspended particulates and heavy metals. The consumption of various fossil fuels is also increasing year by year, and large amounts of aerosols, particulate matter and harmful gases are emitted into the atmosphere. Overall, Chinese industrial development more notably depends on the consumption of fossil energy, during which many harmful pollutants are discharged into the atmosphere^3^. However, the levels of public health services and environmental supervision are unsatisfactory, which has been difficult to control for a long time, resulting in ecological destruction and a threat to human health.

According to statistical data, air pollution significantly increases the chance of the development of respiratory conditions and death resulting from cardiorespiratory causes^4^. High levels of aerosol particles can result in oxidative damage to human DNA and can exert a major negative impact on human health^5^. High ozone levels frequently coexist with high aerosol levels during pollution events, raising additional health concerns^6^. Air pollution imposes obvious and immediate effects on human health, not only increasing the incidence of respiratory and cardiovascular diseases among people^7^ but also affecting the climate and causing more extreme weather events, including floods and droughts^8^. In conclusion, air pollution negatively impacts the lives and health of the Chinese people, deteriorates the natural environment and reduces the sustainable competitive advantages of cities.

Therefore, it is necessary to further analyze of the spatial and temporal attributes of pollutants in the atmosphere and the influencing factors of the air quality and reasonably predict the air quality to provide a level of protection for both humans and nature^9^, improve the urban air quality, create a more livable urban environment^10^, offer a more reliable scientific foundation for the formulation of both environmental and health policies, and provide helpful recommendations, which is also the main focus of contemporary urban development^11^.

In the current literature, the visual method is frequently utilized in analytical studies to perform spatiotemporal visual analysis of air pollution. Shindell et al. studied Arctic air pollution attributed to the flow of atmospheric pollutants in space^12^, and used maps, box plots, and line charts to assist the analysis. Sanderson et al. examined the global flow of airborne nitrogen oxides^13^ in conjunction with the use of maps for visualization-assisted research. Cui et al. used a map to visualize the annual average change in NO_2_ in 341 prefecture-level Chinese cities from 2005 to 2016^14^ and analyzed its temporal and spatial variations, to establish more effective regional emission reduction policies for sustainable development. In regard to spatial analysis, Cai et al. constructed a high-resolution database of Chinese pollutant emissions, thereby laying a solid foundation for the study of Chinese environmental emissions and emission reduction policies^15^. In this study, maps were adopted as visual representations of spatial information. Bennett et al. examined the health hazards of particulate matter with an aerodynamic diameter smaller than 2.5 µm (PM2.5), specifically its impact on lifespan, and they further considered the effect of PM2.5 reduction on the U.S. population^16^. Map and scatterplot visualization methods were employed in this study. In these studies, the visual analysis method was inconsistently used for research, thereby employing a single visualization approach, with no or insufficient interaction analysis. As a result, visual analysis of air pollution has remained at the auxiliary stage. Furthermore, high-dimensional spatiotemporal visual analysis has been less frequently coupled with other potentially associated influencing elements, and concentration variations, impacts, and causes have only been examined for one or more air pollutants.

Qu et al. introduced a comprehensive system for meteorological data visualization combining novel techniques with tried-and-true visualization technologies, including the improved parallel coordinates plot (PCP) with S-shaped axes, circular pixel bar graphs integrated in polar systems, and weighted full diagrams. This method was used to examine the air pollution issue in Hong Kong^17^. Thomas et al. interpreted the content of particles in an air pollution dataset using the PCP method^18^. In this study, they mainly visualized high-dimensional data of air pollutant concentrations and weather conditions. Bachechi et al. conducted dimensionality reduction and spatial clustering of air pollution datasets and then visualized these data in 2D space^19^. Yanosky et al. performed a spatiotemporal analysis of PM2.5 and respirable particulate matter (PM10) with map visualization as the analysis method^20^. Deng et al. studied the propagation patterns of particulate matter in the atmosphere via a visual analysis method^21^. Engel et al. designed an interactive visualization framework for a high-dimensional air quality dataset^22^. Li et al. visualized air quality variation regulations and related meteorological attributes in different cities, thereby revealing the relationship between smog and meteorological attributes^23^. Lu et al. developed two visualization tools for multigranularity time series to study urban air quality data^24^. The air pollution datasets in these studies exhibit high dimensions, and some datasets contain incorporated weather elements with visual analytic methods for analysis purposes, but the influencing factors remain inadequately understood. Moreover, high-dimensional, temporal, and spatial visual analysis research remains rare.

Based on previous research, we employed various visual channels, interactive analysis technologies, and spatiotemporal and high-dimensional visual encoding methods in this study to investigate the potential influencing factors, temporal and spatial variation characteristics, and development trends of air pollution from distinct viewpoints. The primary contributions of this study can be described as follows: (1) creating a high-dimensional spatiotemporal air quality dataset by gathering relevant data and associated meteorological, demographic, economic, and other parameters and (2) performing interactive visual analysis from distinct perspectives. To specifically address the challenges of our air quality dataset analysis approach involving various interactive technologies, the kernel density curve was adopted to expand the histogram for attribute distribution visualization. To reflect the temporal change in attributes, a kernel density ridgeline chart was generated. To detect the relationships between paired attributes, the Pearson coefficient was introduced. (3) We designed and implemented a visual analysis system that can simultaneously analyze high-dimensional, temporal, and spatial data. We demonstrated how to effectively visualize the evolution of multiple dimensional attributes of air pollution data over time and space by using maps, PCPs, and time axes and customizing scatterplot matrices.

The investigation of our interactive visualization technique produced certain beneficial results. First, research and analysis of high-dimensional spatiotemporal datasets could yield promising findings. Moreover, interactive visual analysis of high-dimensional spatiotemporal datasets from multiple perspectives not only produces satisfactory visual effects but also provides a greater display power of data details. Second, in this study, we focused on supporting the visualization and interactive exploration of high-dimensional spatiotemporal air pollution datasets, extending the research methods and fields of previous work, thereby providing assistance to decision-makers in the formulation of air pollution control and health hazard prevention strategies, which has important practical implications for subsequent knowledge-based decision-making processes and auxiliary research.

In this study, we first reviewed relevant spatiotemporal visual analysis, high-dimensional visual analysis, and air pollution studies. Next, the research contents were described in detail, including visualization methods and visual interactive analysis methods. We arranged the air pollution data and related or possibly related factors, organized these data and factors from temporal and spatial perspectives, and finally established a high-dimensional spatiotemporal air pollution dataset. Finally, with the use of the enhanced visual approach in conjunction with the interactive analysis method, we thoroughly examined the established high-dimensional spatiotemporal air quality dataset from various perspectives, obtained certain findings, and formulated recommendations.

## Materials and methods

### Study area

We analyzed the air quality features of 31 Chinese provinces in this work.

### Data source

We used air quality monitoring data of 369 urban areas in the 31 Chinese mainland provinces from January 2014 to December 2021, including the observation time, air quality index (AQI), and ambient concentration of the six main components of PM2.5, NO_2_, O_3_, SO_2_, CO, and PM10, where the concentration unit of CO is mg/m^3^, and that of the other five main components is μg/m^3^. These data are calculated and displayed in https://www.aqistudy.cn/historydata/ and have been compiled monthly since December 2013. There are two reasons for selecting this online air quality dataset. First, the dataset covers a wide time range and a comprehensive range of cities and regions. Second, this dataset is based on a monthly time scale, which is convenient for creating monthly and yearly visualizations, and requires less data calculation.

We also obtained information available to the general public^25^ regarding additional elements that might impact the air quality, including population, per capita disposable income, gross regional product, per capita gross regional product, level of education, urban green area, total afforestation area, fossil energy consumption, natural gas consumption, electricity consumption, secondary industry GDP, and number of civilian automobiles. We collected the above data for 31 provinces in China from 2014 to 2021, and the data were retrieved from the National Bureau of Statistic (http://www.stats.gov.cn/tjsj/).

China meteorological data, i.e., data on meteorological elements, including temperature, wind speed, and precipitation, from 2014 to 2021 were obtained, covering more than 400 stations, and the data were retrieved from the National Climatic Data Center (NCDC) (https://www.ncei.noaa.gov/).

After processing the aforementioned data, a 25-dimensional air quality dataset of China with high-dimensional general and spatiotemporal attributes was created.

### Dataset preparation

Before visualization and analysis, the collected datasets were preprocessed for their direct application to visual analysis tools. We developed a code in the Python language for data cleaning and preparation.

First, we collated the raw air quality data and grouped the selected cities into their respective provinces, and strictly in accordance with the urban scale concentration calculation standards of the evaluation items noted in Ref.^26^, the monthly concentration of each air index in each province was calculated. Then, according to the annual evaluation calculation method, the annual mean value of each index was obtained.

The annual average concentrations of NO_2_, CO, PM10, SO_2_, and PM2.5 in the atmosphere were measured for review. The 90th percentile of the daily maximum 8-h average of O^3^ and the 95th percentile of the 24-h average of CO were used in the annual evaluation process. The percentile can be calculated by arranging the pollutant concentration data in ascending order. The sorted concentration sequence can be denoted as $\{ X_{(i)} ,\;i = 1,\;2, \ldots ,\;n\}$. First, the ordinal number *k* of the *p*th percentile *m*_*p*_ can be obtained as follows:

1 $$\begin{array}{*{20}c} {k = 1 + (n - 1) \times p} \\ \end{array} \%$$

where *k* is the ordinal number corresponding to the *p*th percentile, and *n* is the concentration value in the pollutant concentration series. Then, the *p*th percentile *m*_*p*_ can be calculated as follows:

2 $$\begin{array}{*{20}c} {m_{p} = X_{(s)} + (X_{(s + 1)} - X_{(s)} ) \times (k - s)} \\ \end{array}$$

where *s* is the integer part of *k*, and *s* is equal to *k* when *k* is an integer.

Then, based on the 31 provinces, the AQI for the 12 months of each year was collated, and this dataset was stored in JSON format to facilitate spatiotemporal map visualization. Then, conversion into another format was performed, and the dataset was stored in CSV format to facilitate ridgeline chart generation.

Third, time information, geographic location (latitude and longitude) information, and other attributes related to the air quality were collated and compiled into a comprehensive high-dimensional spatiotemporal dataset, which could facilitate the establishment of maps, PCPs, scatter plots, and data tables.

Finally, a correlation coefficient dataset was generated using the most commonly employed Pearson correlation coefficient^27,28^, via the corr() function in the Python package, thereby excluding any nan values. The Pearson correlation coefficient was denoted as $\rho (x,\;y)$. Then, the Pearson correlation coefficient between two attributes *x* and *y* can be defined as:

3 $$\begin{array}{*{20}c} {\rho (x,\;y) = \frac{cov(x,\;y)}{{\sigma_{x} \sigma_{y} }}} \\ \end{array}$$

where $\sigma_{x}$ and $\sigma_{y}$ are the standard deviation, and $- 1 \le \rho (x,\;y) \le 1$, with a value less than 0 suggesting a negative correlation and a value greater than 0 suggesting a positive correlation.

### Methods

Visual analysis is based on the classical visualization technique and involves the use of interactive methods to explore and analyze complicated datasets^29^. Regarding high-dimensional spatiotemporal data, the visual analysis method is especially suitable for research and exploration. Visual analysis methods can not only consider geospatial information but can also ignore high-dimensional attributes and intuitively express the change and development of variables over time. In particular, different visual interaction techniques can be adopted for rapid and efficient exploration.

#### Spatiotemporal map

**Figure 1:**
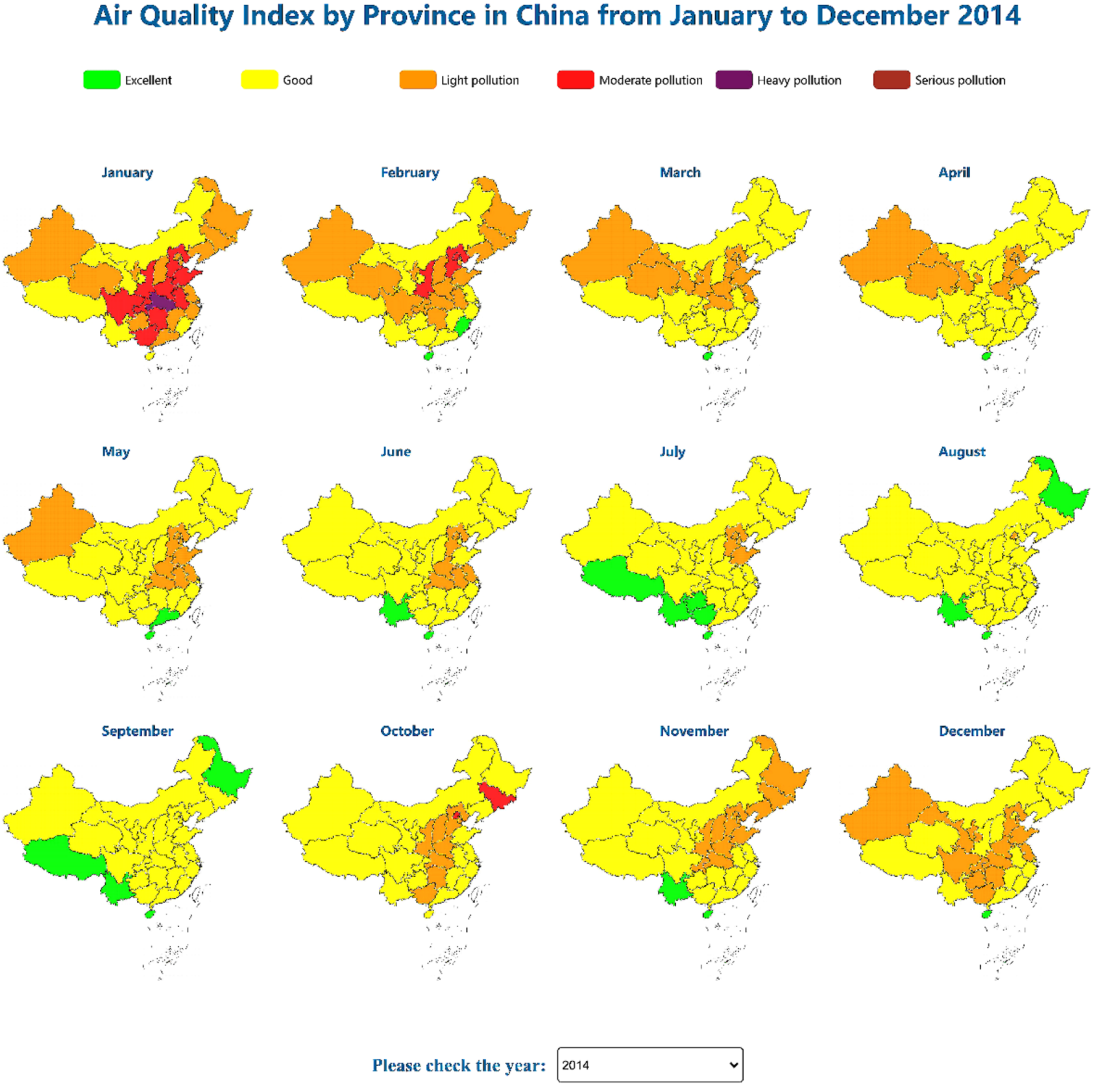
Spatiotemporal maps of the air quality index.

In visual analysis, maps are the most commonly used spatial visualization tools because the presentation of maps enables both professional and nonprofessional analysts to achieve preliminary assessment of the evolution of events in geospatial areas^30^. Accurate AQI forecasting benefits the local economy, environment, and public health^31^. Therefore, we created a spatiotemporal map to reveal the monthly changes and developments in the air quality across the 31 Chinese provinces between 2014 and 2021, thereby employing color channels to display the AQI and a time drop-down list to visualize the time sequence. Moreover, we added highlighting interaction and mouse hover prompt box interactive analysis. The highlighting interaction process here marks the boundary line of the current administrative division in bold to realize the exploration of a single specific value. As a result, overall spatiotemporal exploration could be conducted, and all the details could be grasped, as shown in Fig. 1. A demo of the spatiotemporal map is available at http://18.223.136.39:8080/aqi/page1.html.

#### Kernel density estimation curve

Kernel density estimation is a nonparametric estimation technique^32,33^. In contrast to commonly used parameter estimation methods, such as likelihood estimation, a better model can be obtained by fitting the probability distribution of the dataset according to the data characteristics and properties without adding any prior knowledge. Therefore, in this study, we proposed the use of kernel density curves to visualize air quality datasets. Let *f* be the probability density function of the independent identical distribution $F$ with $n$ sample points $x_{1} ,\;x_{2} \; \cdots \;x_{n}$, in which the kernel density can be estimated as:

4 $$\begin{array}{*{20}c} {\hat{f}_{h} (x) = \frac{1}{n}\mathop \sum \limits_{i = 1}^{n} K_{h} (x - x_{i} ) = \frac{1}{nh}\mathop \sum \limits_{i = 1}^{n} K\left( {\frac{{x - x_{i} }}{h}} \right)} \\ \end{array}$$

where $K( \cdot )$ is a kernel function. There are several varieties of kernel functions, such as uniform and Epanechnikov kernel functions. The smoothing parameter referred to known as the bandwidth must be specified for $h > 0$. $K_{h} (x) = \frac{1}{h}K\left( \frac{x}{h} \right)$ is the scaled kernel. In this study, the Epanechnikov function was chosen as the kernel function, which exhibits the best mean square error and suffers little efficiency loss. The Epanechnikov function can be expressed as follows:

5 $$\begin{array}{*{20}c} {K(x) = f(x) = \left\{ {\begin{array}{*{20}c} {\frac{3}{4}(1 - x^{2} ),} & {\quad \left| x \right| \le 1} \\ {0,} & {\quad otherwise} \\ \end{array} } \right.} \\ \end{array}$$

**Figure 2:**
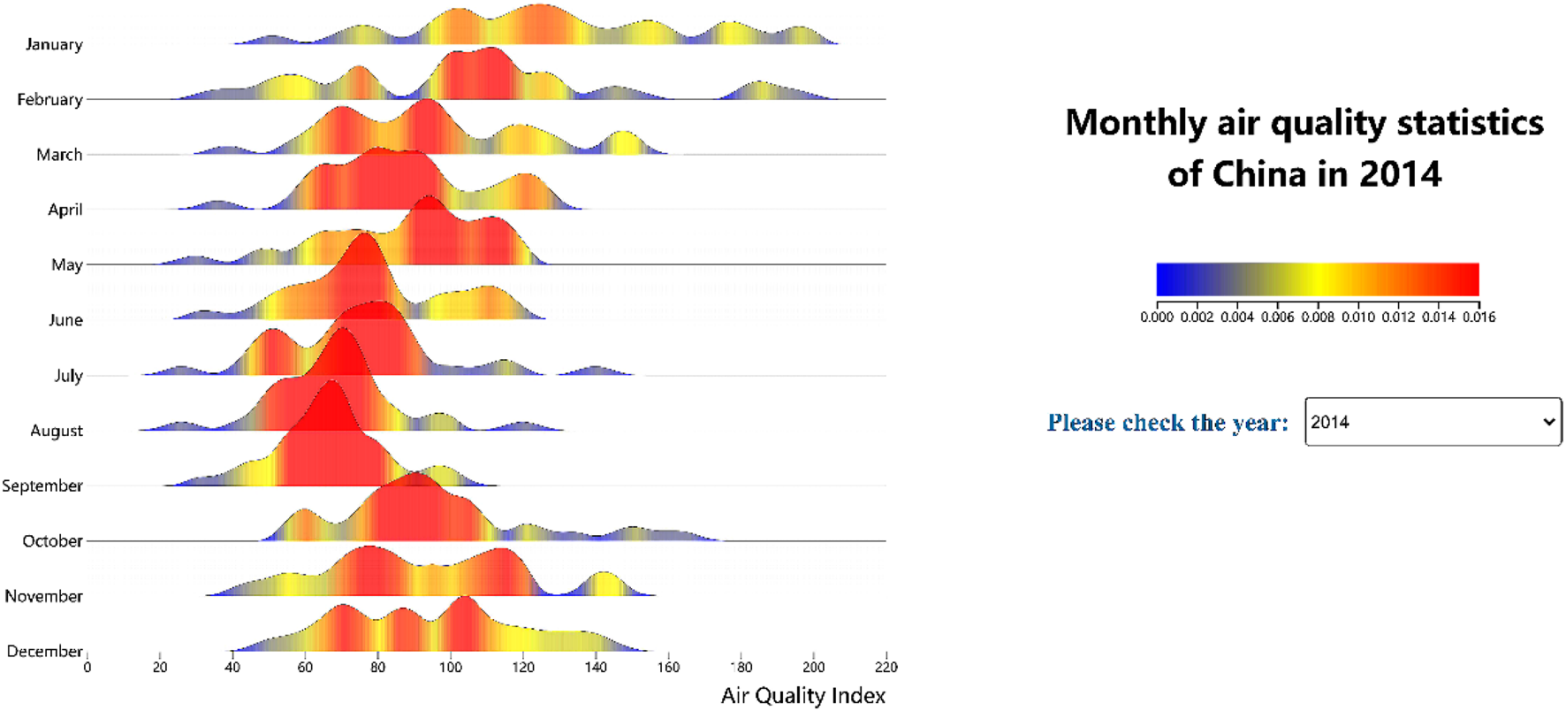
Ridgeline chart of the air quality index.

In this study, we proposed the visualization of the monthly AQI data of each year via a kernel density curve, which can be arranged by month to display the data information of the whole year. Because the shape is similar to that of continuous mountains, the graph is referred to as a ridgeline chart. The properties of the data probability distribution cannot be fully captured by the kernel density curve alone, and the number of visual encoding channels should be kept to a minimum. We added a visual channel involving a continuous color hue to the internal area of the curves to reflect the distribution through a gradual change in colors to produce a visually prominent effect, enhance the numerical expression, and increase the intuitiveness of the visualization effect, as shown in Fig. 2, which is similar to the above spatiotemporal map in which the visual representation of time series is further implemented in the form of a drop-down list. A demo of the ridgeline chart is available at http://18.223.136.39:8080/aqi/page2.html.

In addition, in this study, we used a kernel density curve on the diagonal of the scatter matrix to visualize the numerical distribution of each attribute. Here, this was used with a histogram to display the attribute distribution in a continuous manner.

#### Pearson correlation coefficient matrix

**Figure 3:**
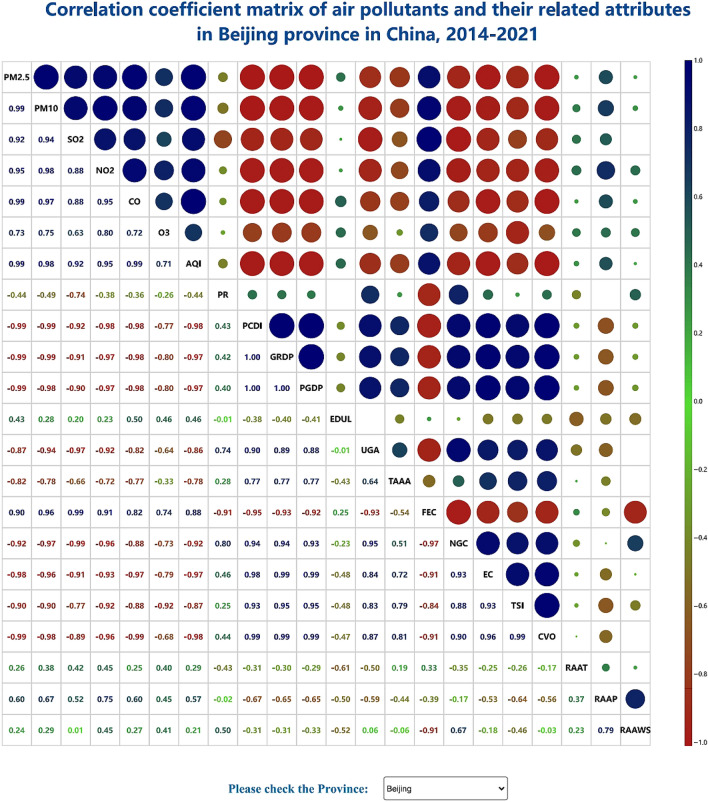
Pearson correlation coefficient matrix.

The correlation is a statistical indicator of the relationship between paired attributes. The correlation coefficient is a statistical metric indicating the degree of connection between variables. A positive correlation suggests that both attributes concurrently increase, while a negative correlation suggests that one characteristic improves while the other deteriorates. There are other ways to define the correlation coefficient, but the most commonly used method is the Pearson correlation coefficient. In the high-dimensional spatiotemporal dataset collated in this study, there are 22 general attributes. To investigate the aspects that influence the air quality, analyze the relationship between the various indicators, and examine the causes of air pollution, we proposed to visualize the correlation coefficients of these 22 dimensions and display them in the form of a matrix. The diagonal line was designed to display attribute names, and the lower triangle of the matrix shows the value of the relationship between the pairwise attributes with a color visual channel. The upper triangle of the matrix cooperatively encodes the correlation coefficient value using the circular area and color visual channels. While the color of the circle indicates the occurrence of a positive or negative correlation and the correlation degree, the size of the circle area reflects the magnitude of the correlation level. This design is succinct, understandable, and effective and adheres to the visual encoding concept. As shown in Fig. 3, the color gradient also serves as a legend to explain the numerical significance of the color channel. A demo of the Pearson correlation coefficient matrix is available at http://18.223.136.39:8080/aqi/page3.html.

#### PCP

For high-dimensional datasets, the PCP^34^ is a popular exploratory analysis and visualization tool. This method expands our capacity to simultaneously display various aspects of high-dimensional datasets in two-dimensional planes and to visualize high-dimensional data across a wide range of applications, as confirmed in a number of disciplines, including computer science^35^, health sciences^36^, and ecology^37^. After the widespread use of PCPs, their inherent defects have been exposed. Therefore, many scholars have also implemented a number of excellent improved techniques, such as highlighting^38^, brushing technology^39^, and axis exchange^40^, which can realize data highlighting, data screening and filtering, and axis sorting according to the importance of attributes, to enhance the PCP visualization performance^41^.

In this work, the 22 high-dimensional generic attributes were also visually encoded with the PCP method. Furthermore, five interactive analytical methods were designed to assist in analysis and exploration. First, in the statistical coloring approach^42^, a certain axis was selected as the benchmark, and the polyline was color coded in accordance with the attribute value. Second, the highlighting method was adopted. When a given polyline is selected, it can be highlighted via thickening with the transparency set to 1, and the position information of the current polyline can be displayed with the tooltip. Third, brushing technology enables users to more clearly comprehend the change trend of local data and better realize the study of data features by highlighting the chosen polyline portion while hiding other polylines. Fourth, the coordinate axis exchange technique was implemented. This could allow analysts to tentatively change the order of axes and compare the attributes at adjacent positions to obtain the internal relationship, rules and implicit trends between two attributes. Fifth, axis reverse technology was applied to display polylines in a satisfactory and easy-to-observe form, rather than in the form of crosses. The attribute name above the coordinate axis displayed in reverse order was underlined.

**Figure 4:**
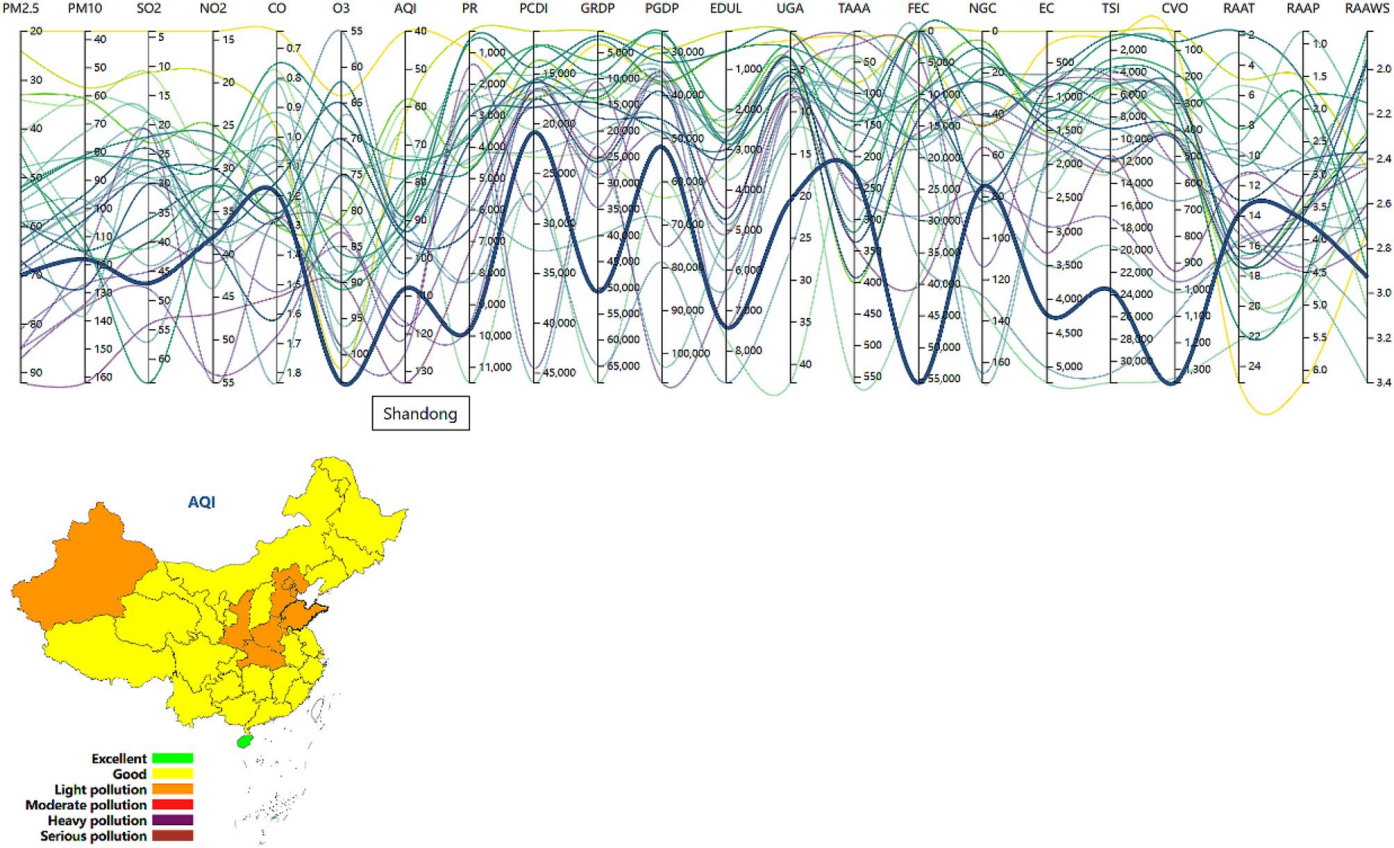
Interrelated highlighting interactions.

In addition, in joint visual analysis of PCPs and maps, we designed a linkage interaction analysis approach. The map and line were concurrently highlighted, and the corresponding administrative divisions in the map could be selected while the line is highlighted, as shown in Fig. 4. By clicking the attribute name above the coordinate axis, axis reversal could be realized, while the map color indicates the value of the current attribute, and the corresponding legend is displayed.

#### Scatterplot matrices

**Figure 5:**
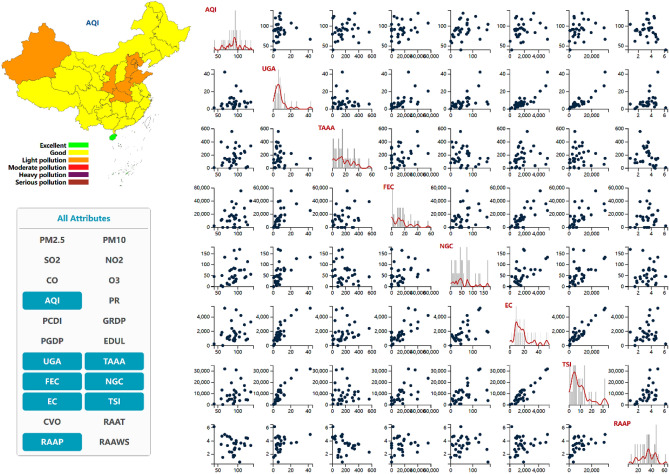
Scatterplot matrices of the customized attributes.

The scatterplot matrix^43^ is a popular visualization method used to display the relationship between pairwise dimensions. The air quality dataset in this study contains a wide range of attributes. This may result in display congestion and confusion if presented in a constrained interface in the form of a scatterplot matrix. Therefore, we designed a scatterplot matrix that can be customized in terms of attributes. Notably, we selected several attributes in the left attribute list to generate a corresponding scatterplot matrix. The number of attributes is controlled within 8, and the effect is improved, as shown in Fig. 5.

The diagonal of the scatter matrix is a very efficient way to show the overall distribution of each attribute using a histogram^44^. To reflect the data distribution more objectively and better, it is necessary to reasonably set the value of the main parameter bins. Here, we achieved better results by setting the bin value to 40. However, the histogram is affected by its inherent defects, the distribution is not smooth, the shape is discontinuous, and different bin parameters can produce varying visual effects. To solve this problem, a one-dimensional kernel density estimation curve was added to the histogram in this study to visualize the distribution of attributes together with the histogram. The main parameter of the kernel density curve, i.e., the bandwidth, was set to 3.

We also added a scaling factor to the data value so that the histogram and kernel density curve could be effectively expressed together. Due to the various dimensional units, the data of the various dimensions exhibit various scaling factors, as shown in Fig. 5, and the kernel density curve captures the effect after scaling.

#### Tables and software package

The visualization methods involved in this study, including PCPs, scatterplot matrices, kernel density curves, histograms, and maps, were all implemented using the D3.js visualization library^45^. D3.js is a notable database and uses a scalable vector graphics file format to generate high-resolution web graphics pages, thereby providing a high customization degree, which is suitable for the customization of various types of visualization needs and visual interaction technologies.

**Figure 6:**
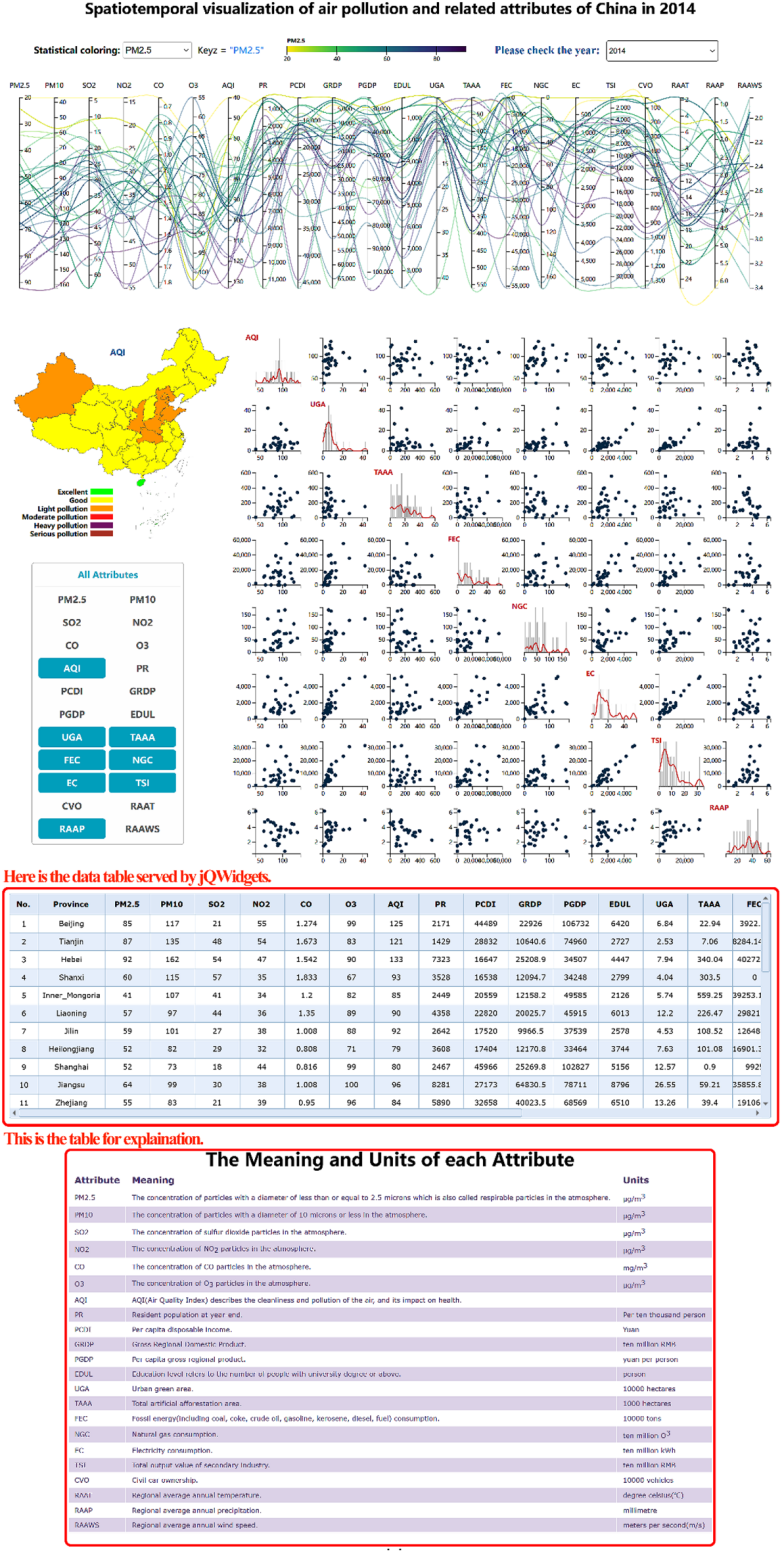
Comprehensive visualization of the high-dimensional spatiotemporal air quality dataset. PCPs, maps, customized scatterplot matrices, and assisted tables are integrated. It is easy to visually analyze spatiotemporal datasets from various fields through interactive analysis methods.

Moreover, D3.js provides a color scheme, which can mitigate the notable visualization problems caused by self-color matching, and the color scheme is very flexible. In addition, the grid of the free and open-source jQWidgets framework^46^ served as table support to visually display the organized air quality dataset, as shown in Fig. 6. The visualization and interaction analysis results were finally realized in the form of web pages in this study.

## Results

### Spatiotemporal analysis of the AQI

The AQI is a nondimensional indicator used to describe air quality conditions, and varying air quality levels are associated with different health implications. According to Ref.^47^, the AQI can be split into five categories from 0 to 300, which reflect excellent, medium, light, moderate, and severe pollution, with values exceeding 300 denoting dangerously polluted conditions, and corresponding color values were used to represent the various levels. These values could allow us to assess the air pollution degree and implement the appropriate precautions to protect people.

We initially used the map visualization approach to create a spatiotemporal map of the AQI for each month of the whole year from 2014 to 2021 to better understand the spatiotemporal variation in the air quality. With the provincial administrative division classification as the fundamental analytical unit, each administrative division was color filled according to the recommended grading color, and the time axis was used to visualize the time series, as shown in Fig. 1. The interactive analysis method involving highlighting and tooltip use was employed for exploration, and the legend of the color channel was provided above the map.

The spatiotemporal map of the air quality revealed that the air quality was generally best in summer and worst in winter, which may be related to winter heating, and the air quality in spring and autumn was generally average. It could be concluded that the air quality was largely related to seasonal variations. Analysts can also select the year of choice from the drop-down list to observe the AQI changes for each year. We also discovered that the air quality is improving every year, which may be due to various causes, including governmental energy-saving and emission reduction initiatives, the usage of new clean energy cars, and afforestation.

To evaluate the air quality changes in China as a whole, in this study, we also introduced a one-dimensional kernel density curve to visualize the distribution of the AQI in the 31 administrative divisions, which was arranged by month to generate a ridgeline chart with continuous colors according to the AQI distribution. The bandwidth parameter was set to 7. As shown in Fig. 2, the higher the kernel density curve is, the higher the density, i.e., the occurrence probability is higher. We colored the internal area red. In contrast, the lower the curve is, the lower the occurrence probability, and the internal area was colored blue. The color legend is shown on the right side. The color is continuous and gradually varies with the change in the density value. The color channel was used to encode the inner area of the kernel density curve, which is crucial for revealing the density.

The X-axis of the figure denotes the AQI value, which gradually increases from left to right, indicating that the air quality is decreasing. In the figure, a red part can be observed on the left, and the larger the red area is, the better the air quality. Analysts can change the time from the drop-down list to observe the temporal evolution of the data, and it could be easily found that the red area increased each year and was located farther to the left, further verifying that the air quality is improving year by year.

### Correlation visual analysis of air pollutants and other related influencing factors

The administrative division was chosen as the unit of the collated high-dimensional spatiotemporal dataset in this study, and the values of each dimension from 2014 to 2021 were obtained to calculate the Pearson correlation coefficient value, which was visualized as a matrix, as shown in Fig. 3. Positive and negative correlations were indicated by various colors, and the size of the circular area could be used to convey the connection degree. Red denotes a negative connection, while blue denotes a positive association. The strength of the connection increases with increasing absolute value of the correlation coefficient. If the value is 0, irrelevance is indicated, and the transition color green is used in the middle. Here, the circle area and color channels were used as the key encoding channels to reveal the correlation between attributes. The value and color of the lower triangle were used to help explain the specific related situation.

With the use of Beijing as an example, it could be readily observed that there exists a substantial link between pollutants and air quality, with fossil fuel use as the dominant factor. It could be concluded that the burning of fossil fuels is the primary source of atmospheric pollution. There is also a certain positive correlation between the air quality and rainfall. In other words, wet days could aggravate air pollution and prevent dangerous particles from dispersing into the atmosphere. The air quality was also negatively correlated with the urban green space area and the artificial afforestation area to a large extent. Notably, afforestation could effectively reduce air pollution. Therefore, air pollution could be effectively controlled and prevented by increasing the green area. Moreover, natural gas and electricity could be used as clean energy to reduce air pollution occurrence.

### Multidimensional spatiotemporal data visual analysis

Figure 6 shows the comprehensive visual analysis interface of the high-dimensional spatiotemporal air quality dataset. PCPs were used to display the high-dimensional general attributes, a map was used to visualize the geospatial attributes, and the time sequence was displayed through the drop-down list. Scatterplot matrices, histograms, and kernel density curves were used to assist in realizing the visualization system of the high-dimensional spatiotemporal dataset.

The combination of maps and PCP is a very effective, verified, and general visual analysis method for geospatial high-dimensional data, such as geo-coordinated parallel coordinates (GCPC)^48^. To better comprehend the distribution and association of features, the combination of scatterplot matrices and PCPs has been frequently used to depict high-dimensional information^49,50^.

On the basis of previous work, we proposed the combined visualization of maps, PCPs and scatterplot matrices, in addition to a time axis, which could simultaneously realize high-dimensional, temporal, and spatial data visualization. In addition, we designed joint interactive analysis techniques, which could be applied to better understand the influencing factors and development trends of the air quality to offer a scientific foundation for air pollution management and ecological environment improvement.

At this stage, PCPs could provide visualization and interactive analysis of high-dimensional general attributes. Interactive technologies could help users find information and facilitate the exploration process. To promote overall data observation, the drop-down option allows us to change the attribute axis of the statistical coloring scheme in this case. Second, the highlighted polylines, highlighted map administrative divisions and prompt boxes generated via mouse hovering could elucidate the association between the high-dimensional attributes and geographic information by sensing the implicit connections generated by all highlighted matches. Analysts can start exploration from high-dimensional attributes as well as geospatial attributes to explain the situation of atmospheric pollutants and other factors that may exhibit a causal relationship with these pollutants. To facilitate the comparison of pairwise attributes, the axis exchange interaction analysis method and axis reverse interaction method were used in PCP generation to analyze the data more conveniently and quickly. By clicking the attribute name above the coordinate axis, the data value displayed in the map can be switched to the current attribute, combined with the corresponding legend. Users can filter data using brush techniques^51^.

Integrating other visualization methods with the PCP technique could facilitate the visual information-seeking process by revealing different data aspects that are difficult to reveal through polyline patterns alone. Scatter plots are a popular visualization method to evaluate whether there exists a correlation between two variables.

We established an attribute customization method for the scatter graph matrix due to the high dimension and constrained page coverage. Through selection interaction, the selected attributes can be displayed in the resulting scatterplot matrix.

As shown in Fig. 6, the selected attributes include PM2.5, AQI, UGA, TAAA, FEC, NGC, and EC. In this chart, the distribution of points in each scatter plot and the general trend of variable change can be examined.

On the diagonal, the histogram and kernel density curve jointly show the distribution of the individual attributes to help users more accurately perceive the data occurrence frequency. Finally, the raw data can be visualized in the form of a table, so that analysts can intuitively analyze these data to assist assessment, as shown in Fig. 6.

Additionally, we attached a table below to explain the abbreviations of each attribute, as shown in Fig. 6. A demo of the proposed high-dimensional spatiotemporal air quality dataset visual analysis method is available at http://18.223.136.39:8080/aqi/page4.html.

The time drop-down list can be switched to visualize and analyze the evolution of each attribute over time. By observing the polyline changes, we could track the spatiotemporal variation characteristics, spatial distribution characteristics and variation trends of the air quality and found that the primary pollutants affecting the air quality differed in each province, which may be closely related to the local industrial structure, climatic conditions, and geographical location to a certain extent, among which wind was more conducive to the diffusion of air pollutants. Moreover, we found that air pollution prone areas were geographically consistent with high fossil energy consumption levels, such as Shandong Province and Hebei Province. The use of map visualization to achieve comparison among the various administrative division units could play a favorable role in correlation analysis because air pollution is not restricted by regional boundaries, let alone national boundaries. Through visualization, it could be possible to estimate the degree of responsibility of polluters. Clicking the attribute name on the parallel coordinate axes can quickly switch the attributes displayed on the map, allowing us to better understand the spatial distribution of the different attributes from a spatial perspective, which is helpful for developing strategic strategies for air pollution reduction efforts.

Overall, it is challenging to fully visualize and interactively analyze high-dimensional spatiotemporal information. Our design could help governments better understand the causes of the spatiotemporal distribution, and potential influencing factors of air pollution so that they can formulate and implement important action plans to curb its development.

## Discussion

Air pollution poses notable hidden risks to the global ecology and exerts potential long-term impacts on human health and social development, which it has further aroused the attention of academia and policymakers. Real-time air quality and pollution monitoring information has been made available by the China Ministry of Environmental Protection since January 2013. It is now feasible to research the temporal and spatial aspects of air pollution across China owing to the development of monitoring infrastructure and continual extension of the monitoring scope^52^. In this study, we used 8 years (2014–2021) of air pollution data for the provincial administrative divisions in China and high-dimensional spatiotemporal data comprising related factors and proposed a comprehensive integrated high-dimensional spatiotemporal air pollution data visual analysis system using various visual encoding methods and interactive analysis technologies to comprehensively explore the data from multiple perspectives.

The Chinese government has gradually implemented different emission control measures with a focus on the use of renewable energy and the reduction in industrial and vehicle exhaust emissions^53^. Additionally, comparable emission reduction goals have been developed based on the unique characteristics of each province^54^. The Chinese government is actively promoting the use of technology for emission reduction and clean energy at the same time. Studies have demonstrated that clean energy technologies can indeed greatly improve the air quality^55^. This further verifies the reasons for the improvement in the air quality in China, and data analysis could provide guidance for air pollution control measures. In this study, we illustrated the relevance of our results for policymaking purposes.

## Conclusions

From this research, the following findings could be drawn:

1. The air pollutants in each region vary, and the causes also differ. Therefore, governance must be targeted and adapted to local conditions.
2. Vegetation and terrain can affect the air quality. Vegetation can absorb pollutants and improve the air quality. However, excessively dense vegetation can also affect the diffusion of pollutants to a certain extent.
3. There exists a strong self-correlation between air pollutants, and the accumulation of various pollutants can aggravate the decline in the air quality. Therefore, it is necessary to coordinate governance in formulating countermeasures and scientifically guiding the direction of policies.
4. Wind can accelerate pollutant dispersal and diffusion, resulting in a decrease in the concentration. However, rainy weather can unfavorably impact the spread of pollutants to a certain extent.
5. The air quality exhibits a certain seasonality, and the air quality is generally poor in winter, which is related to the consumption of fossil fuels for heating purposes. Therefore, clean energy is an inevitable future development trend.

The air quality is a topic of great concern. In years affected by smog, people wear masks during travel, which affects traffic, tourism, urban construction, etc., and causes a surge in the number of pathologies.

Data research and remedial measures after disasters are far from sufficient to compensate for the losses due to disasters. The high-dimensional spatiotemporal visual interactive analysis approach suggested in this study may offer a theoretical foundation for additional research of a similar nature, serve as a foundation for the development of related policies targeting emission reduction and energy conservation, and offer practical preventive measures in urban planning. Moreover, at the beginning of the construction and design of various systems in local cities, disaster recovery functions should be accordingly planned to establish a solid line of defense for the protection of the lives and property of people.

## Data Availability

The data we utilized in this study are all from public data sources. The air quality observation dataset downloaded from the website https://www.aqistudy.cn/historydata/. The weather in China dataset downloaded from the website https://quotsoft.net/air/, which is from the National Climatic Data Center (NCDC) (https://www.ncei.noaa.gov/). Data on other relevant factors of China downloaded from the National Bureau of Statistics website (http://www.stats.gov.cn/). The datasets we used in our work are all available on Github (https://github.com/liujia120103/air-quality-datasets). The following is a detailed description of each dataset. The original datasets we collected are available on https://github.com/liujia120103/air-quality-datasets/tree/main/original%20datasets. The preprocessed results from these datasets are subsequently used in our visualization studies. The dataset of spatiotemporal maps of air quality index which is shown in Fig. 1 is available on https://github.com/liujia120103/air-quality-datasets/tree/main/1.spatiotemporal_maps. The dataset of ridgeline chart of the air quality index which is shown in Fig. 2 is available on https://github.com/liujia120103/air-quality-datasets/tree/main/2.ridgeline. The dataset of Pearson correlation coefficient matrix which is shown in Fig. 3 is available on https://github.com/liujia120103/air-quality-datasets/tree/main/3.Pearson_correlation_coefficient_matrix. The dataset of high-dimensional spatiotemporal air quality we organized which is served by jQWidgets as shown in Fig. 6 is available on https://github.com/liujia120103/air-quality-datasets/tree/main/4.air_quality_dataset. The dataset of the meaning and units for each attribute in high-dimensional spatiotemporal air quality dataset which is shown at the bottom of Fig. 6 is available on https://github.com/liujia120103/air-quality-datasets/tree/main/5.attribute_meaning. In addition, Figs. 4 and 5 are part of Fig. 6. Besides, there are two tables display in Fig. 6. The first table is a kind of visualization way to show all the records of air quality datasets we organized, which is displayed for assistance. The second table titled “The meaning of units of each attribute” is shown for explaining abbreviations of each attribute. These two tables are all part of our visualization webpage.
